# Direct Current Electrical Performances of Cable Accessory Insulation EPDM Modified by Grafting Polar-Group Compound

**DOI:** 10.3390/polym14214625

**Published:** 2022-10-31

**Authors:** Zhong-Yuan Li, Wei-Feng Sun, Jian Zhang, Jian-Quan Liang, Lei Wang, Ke-Xin Zhang

**Affiliations:** 1 Electric Power Research Institute, State Grid Heilongjiang Electric Power Co., Ltd., Harbin 150030, China; 2 School of Electrical and Electronic Engineering, Nanyang Technological University, Singapore 639798, Singapore

**Keywords:** power cable accessory, polar-group molecule, rubber reinforce insulation, electric conduction nonlinearity, dielectric breakdown strength

## Abstract

In order to improve electrical matching between ethylene-propylene-diene misch-polymere (EPDM) reinforce insulation and crosslinked polyethylene (XLPE) main insulation in direct current (DC) cable accessories, the glyceryl monooleate (GMO) organic compound composed of several polar-groups and one long carbon chain is employed for chemical graft modification on EPDM to ameliorate DC electrical performances. Charge trap characteristics are analyzed by testing thermal stimulation current (TSC) and verified by calculating first-principles electronic properties to elucidate the GMO-graft-modified charge trapping mechanism accounting for DC electric conductance and dielectric breakdown. The grafted GMO molecules introduce substantial shallow charge traps that lead to nonlinear profiles of electric conduction versus electric field and cause hopping transports of percolation conductance. Electric conductance of EPDM is significantly improved by GMO graft for electrical matching with XLPE, while a high level of dielectric breakdown strength is retained sufficiently for reinforce insulation in cable accessories. Shallow charge traps introduced by GMO graft are capable of capturing charge carriers to form homocharge layers near electrodes which can scatter the transporting charge carriers and exclude further charge injections, thus to mitigate the dielectric breakdown strength reduction caused by electric conductivity improvement. Electric field finite-element simulations demonstrate that the electric field in DC cable terminals can be evidently homogenized by using GMO-grafted EPDM as reinforce insulation.

## 1. Introduction

Ethylene-propylene-diene misch-polymere (EPDM) is comprehensively used as an assistant insulation material in high voltage direct current (HVDC) cables due to its excellent dielectric and thermal-mechanical properties, which are appropriate to reinforce insulation in cable accessories. In the multi-layer structure of cable accessories, which is the most weak component in power transmission cable system, the considerable discrepancy in electric conductivity between two adjacent dielectric layers leads to electric field distortion and causes space charge accumulations so as to initiate partial discharges at dielectric interfaces, especially between crosslinked polyethylene (XLPE) main insulation and EPDM reinforce insulation, which will eventually develop into insulation failures of cable accessories [1,2].

Polymer dielectric composites made by filling inorganic nanoparticles into polymer dielectrics have been comprehensively exploited to improve dielectric performances of polymer insulation materials, which is essentially attributed to the generally recognized charge trapping mechanism of inhibiting space-charge accumulation and impeding electric conduction [3,4,5,6]. Inorganic fillers (SiC, ZnO, carbon black, graphite, etc.) in nonlinear dielectric composites especially give rise to a nonlinear dependence of electrical conductivity or dielectric permittivity on electric field strength, as described by “electric conduction nonlinearity” [7,8,9,10,11,12,13]. Nevertheless, traditional inorganic/polymer composites with electric conduction nonlinearity requiring for high filling contents lead to remarkable degradation in electric-breakdown resistance and processing feasibility.

Recently, chemistry modifications of grafting polar-group molecules to improve insulation performances of polymer dielectrics have gained unprecedented attentions, which is realized by a minimal graft content without introducing substantial impurities or degrading polymer processability. By grafting maleic anhydride (MAH) to polypropylene (PP) molecular-chains, a sufficient amount of uniformly distributed deep charge traps can be introduced into PP matrix, accounting for impeding charge carrier transport and raising charge injection barrier, which results in significant improvements of electric resistance and DC breakdown strength [14]. By means of UV-initiated crosslinking technique to avoid the scorched substance, described as an “amber color”, that will severely deteriorate cable insulation, the chloroacetic acid allyl ester (CAAE) was successfully grafted onto polyethylene through UV-irradiated crosslinking process [15]. Carbonyl polar-groups of the grafted CAAE render substantial deep charge traps to effectively inhibit space charge accumulation, decrease electric conduction current, and improve dielectric breakdown resistance. Chemical modifications of grafting MAH and acrylic acid derivatives can significantly improve dielectric breakdown strength and give rise to a distinct nonlinearity in electric conduction characteristics. The other prototype of organic molecules used for chemical graft modifications on polymer insulation materials are composed of specific polar-groups bonding to benzene rings, described as a voltage stabilizer, such as 4-propylene oxyxy-2-hydroxydibenzenone, and has been suggested to dissipate kinetic energies of thermal carriers for restricting electric-tree growth in polyethylene materials [16].

To date, the studies on polar-group graft modifications to improve DC electrical performances in polymer insulation materials have primarily focused on semi-crystalline polyethylene and polypropylene. However, for rubbery amorphous phase materials, it is impossible to ameliorate material properties by ameliorating crystalline morphology or crystallinity, and the other modification mechanisms should be emphasized. Therefore, in the present study, an organic compound—glyceryl monooleate (GMO)—is adopted for chemical graft modification on EPDM, which possesses multiple polar-groups and one long carbon backbone chain in favor of introducing shallow charge traps and preventing thermal migration out of polymer matrix, respectively. Electric conductance and dielectric breakdown strength are focused to improve electrical matching between EPDM and XLPE in cable terminals while persisting a sufficient reinforce insulation strength. First-principles electronic property calculations and thermal stimulation current analyses are performed to elucidate charge trap characteristics introduced by GMO graft, which accounts for charge carrier transport and dielectric breakdown of GMO-grafted EPDM (EPDM-g-GMO) material. Conforming to 200 kV voltage engineering applications, the electric-thermal coupling finite-element simulations on cable terminals are performed to verify EPDM-g-GMO competence as reinforce insulation for homogenizing electric field in cable accessories.

## 2. Experimental and Theoretical Methods

### 2.1. Material Preparation

**Figure 1:**
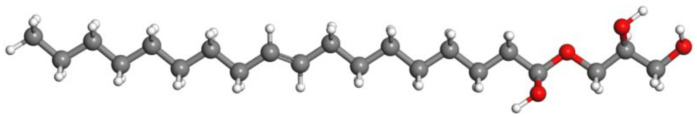
Schematic GMO molecule structure, with the gray, red and white spheres representing carbon, oxygen and hydrogen atoms respectively.

With melting blend and hot press methods, the graft modified EPDM material is prepared with pristine EPDM (4725P, American DuPont Co., Ltd., Chicago, IL, USA) as parent material, the dicumyl peroxide (DCP, Nobel Co., Ltd., Aksu, China) as a crosslinking/grafting initiation agent, and the glyceryl monooleate (GMO, Sinopec Company Ltd., Beijing, China) as graft modification agent (as shown by schematic molecular structure in Figure 1): (1) EPDM raw material is hot-degassed in vacuum oven at 60 °C for 24 h to eliminate moisture; (2) the hot-degassed EPDM with 0.5 phr GMO and 2.0 phr DCP are mixed into torque rheometer (RM200C, Hapro Co., Ltd., Harbin, China) with a rotating rate of 60 r/min, being blended at 100 °C for 10 min; (3) the prepared blend material is pressed to melting under 15 MPa at 110 °C for 15 min in plate vulcanizer and then heated to 175 °C with a heating rate of 5 °C/min and persisting for 30 min, during which crosslinking and grafting reactions are realized; (4) the crosslinked material is naturally cooled down to room temperature, and then hot-degassed at 80 °C for 3 days in vacuum oven to relax thermal stresses and remove residual molecular by-products.

### 2.2. Infrared Spectroscopy

Infrared absorption spectra are tested in wavenumber range of 500~4000 cm^−1^ with a resolution of 2 cm^−1^, as implemented by Fourier-transform infrared (FTIR) spectrometer (FT/IR-6100, Jiasco Trading Co., Ltd., Shenyang, China) for the prepared materials individually with and without passing through the crosslinking process through which GMO grafting reaction is simultaneously realized. Chemical graft of GMO onto EPDM is characterized by contrasting infrared absorption peaks of GMO chemical groups before and after crosslinking (grafting) reaction.

### 2.3. Differential Scanning Calorimetry and Thermogravimetrics

Differential scanning calorimetry (DSC) is adopted to test heat-flow through 10 mg samples of graft-modified EPDM material in reference to EPDM benchmark, when being gradually heated/cooled by a rate of 5 °C/min under nitrogen atmosphere and 1 atm pressure in temperature range of 0–90 °C passing through phase transition between glassy and elastomeric states of EPDM materials, as implemented by differential scanning calorimeter (DSC822e, METTLER TOLEDO, Zurich, Switzerland). Thermogravimetric analysis (TGA) is performed, as implemented by synchronous thermogravimetric analyzer (TG209F1-Libra, NETZSCH Co., Ltd., Berlin, German), to evaluate mass loss in heating process from 100 to 500 °C with a heating-rate of 5 °C/min.

### 2.4. Electric Conductance

Electric conduction characteristics are profiled by testing electric current density versus electric field strength at diverse temperatures of 30–70 °C, as implemented in a standard three-electrode system consisting of DC high voltage power (Keithley 2290-10, Tektronix Co., Ltd., Biaverpton, OR, USA) and electric current meter (EST-122, Huace Co., Ltd., Beijing, China). The tested materials are fabricated into 100 × 100 mm^2^ square film samples of 0.2 mm thickness. A cylindrical copper column of 50 mm diameter is used as the testing electrode to detect current signal; high voltage electrode is an aluminum plate of 78 mm diameter in contact with DC high voltage power supply; protection electrode is a copper ring column with 76 mm outer diameter and 54 mm inner diameter. Before applying voltage, the three-electrode test system and sample are heated in oven for 5 min to attain thermal equilibrium in sample and electrode at each objective testing temperature. Stable conductance current is measured after applying DC voltage for 40 min at each testing point of electric field strength by a step-up of 5 kV/mm in 5–45 kV/mm range.

### 2.5. Thermal Stimulation Current

Thermal stimulation depolarization current versus temperature is tested to analyze energetic distributions of charge traps, as implemented in the temperature-controlled dual-electrode system (TSC, Harbin University of Science and Technology, Harbin, China). The tested film samples of 100 ± 10 μm thickness are polarized by applying electric field of 40 kV/mm for 30 min at 50 °C and then promptly cooled down to −50 °C persisting for 5 min in liquid nitrogen. Depolarization currents are continuously measured on short-circuiting samples whilst raising temperature from −50 to 170 °C with a heating rate of 3 °C/min.

### 2.6. Electric Breakdown Experiment

Dielectric breakdown strength is evaluated by measuring DC electric-breakdown voltages at a diversity of temperatures from 30 to 70 °C. The tested materials are fabricated into circular film samples of 50 mm diameter and 0.15 mm thickness. Asymmetric columnar electrodes of 25 and 70 mm diameters are used for high voltage and ground electrodes, respectively. At the end of the continuously increasing electric field at a rate of 4 kV/s, the maximum voltage recorded just before dielectric breakdown is granted as breakdown voltage. To prevent creepage discharge on sample surface, both the sample and electrodes are immersed in dimethyl silicon oil during voltage applying process. For each material and testing temperature, electric breakdown experiment is repeated for 11 times, with the results being fitted by two-parameter Weibull statistics to evaluate dielectric breakdown strength.

### 2.7. Electric-Thermal Coupling Simulation

**Figure 2:**
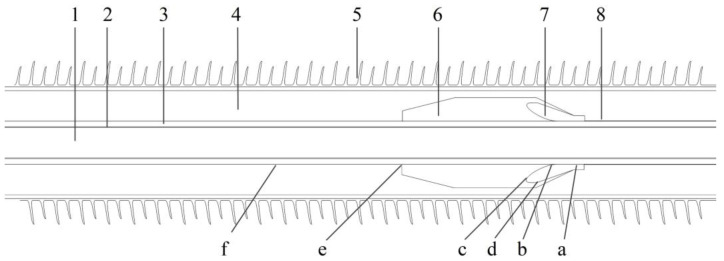
Geometry model of HVDC cable terminal: 1—conductive core; 2—inner shield; 3—XLPE main insulation; 4—silicone oil; 5—ceramic tube; 6—reinforce insulation; 7—stress cone; 8—outer shield. a—stress cone front terminal; b—stress cone root; c,d—stress cone interface; e—common point of main insulation, silicone oil and reinforce insulation; f—cable outer shield.

HVDC cable terminal on 200 kV voltage level composed of EPDM or EPDM-g-GMO reinforce insulation, XLPE main insulation and silicone-oil-filled insulation is modeled according to geometries and dimensions as illustrated in Figure 2 and Table 1. The entire length of cable terminal model is specified as 2600 mm, around which air ambient domain is also specified by a convection heat transfer of 10 W/(m^2^·K). Conductive core is applied by DC high voltage, and internal edge of semi-conductive outer shield is grounded. The temperatures of conductive core and ambient air domain are set as constant values of 70 and 20 °C, respectively.

Material properties of XLPE main insulation and inner shield layers in the present electric-thermal coupling simulations are listed in Table 2 [17]. Since the present study focuses on DC electrical performances of EPDM used for DC cable accessories that are simulated under coupling physical loads of electrostatic field and heat transfer, the relative dielectric permittivity under DC electric field is specified for XLPE main insulation and inner shield layers, as shown in Table 2. With Delaunay triangulation algorithm, the free triangular elements are used for finite-element meshing by an element growth rate of 1.5 and narrow region relaxation of 1.0, which are further refined at the locations where electric field strength and temperature vary dramatically in cable terminal model. The maximum and minimum numbers of triangular elements are adjusted until no obtuse angle arises.

### 2.8. Molecular Modeling and First-Principles Schemes

Electronic properties of EPDM, GMO and EPDM-g-GMO are calculated with first-principles all-electron atomic-orbit schemes, as implemented by DMol3 module of Materials Studio package (Accelrys Inc., Materials Studio version 2020.08, San Diego, CA, USA). Initial polymer models in 30 polymerization degrees of EPDM and EPDM-g-GMO and the molecular model of GMO are constructed by rotational isomeric state method [18], which is geometrically optimized to approach atom relaxations by conjugate gradient algorithm under iteration tolerance of 1.0 × 10^−5^ eV/atom energy, 0.03 eV/Å atomic force and 0.001 Å atom displacement. Atomic projected density of electronic-states and other electronic properties of electron affinity, ionization potential and energy gap are calculated to elucidate the shallow charge traps and the exacerbated electron impact ionization deriving from GMO graft.

## 3. Results and Discussion

### 3.1. Molecular Group Characterization

**Figure 3:**
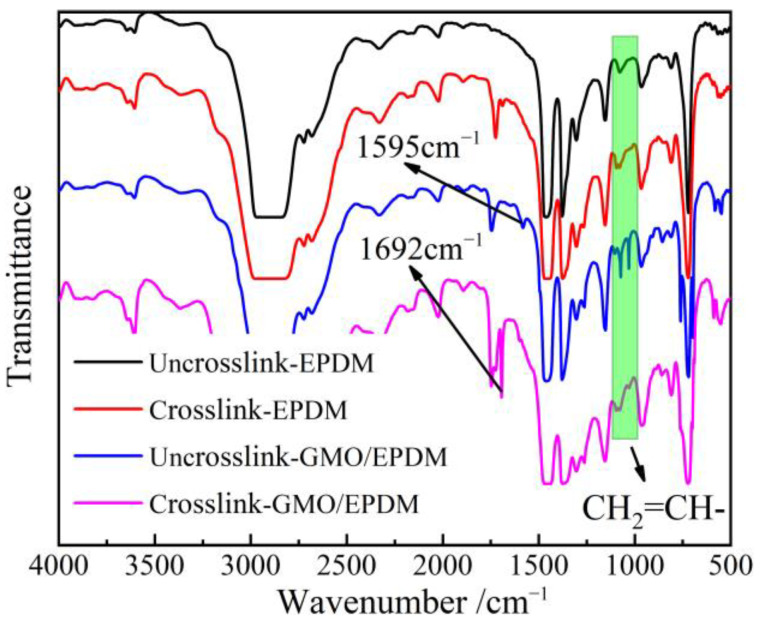
Infrared transmission spectra of EPDM and GMO/EPDM blend before and after crosslinking process.

As shown by infrared transmission spectra of EPDM materials from blend to crosslinked EPDM-g-GMO in Figure 3, the ungrafted GMO molecules containing an alkene double bond (C=C) are characterized by the infrared absorption peaks in 800–1100 cm^−1^ range from the in-plane bending vibrations of C-H bonds adjacent to C=C, which decline notably after crosslinking process.

The pure EPDM after crosslinking process shows a characteristic absorption peak at 1731 cm^−1^ of the saturated ketone group (C=O). The GMO/EPDM blend, without passing through crosslinking process, gives rise to an characteristic peak of C=C at 1595 cm^−1^, which vanishes after crosslinking process, in contrast to the newly appeared 1692 cm^−1^ peak derived from C=O of carboxylate group. Infrared spectroscopy indicates that the C=C and C–H bonds of GMO molecules accounting for grafting reaction disappear after crosslinking process, while retaining C=O groups that cannot participate into grafting reaction, which demonstrates the successful GMO graft onto EPDM by radical addition reaction.

### 3.2. Glassy–Elastomeric Phase Transition and Thermogravimetrics

**Figure 4:**
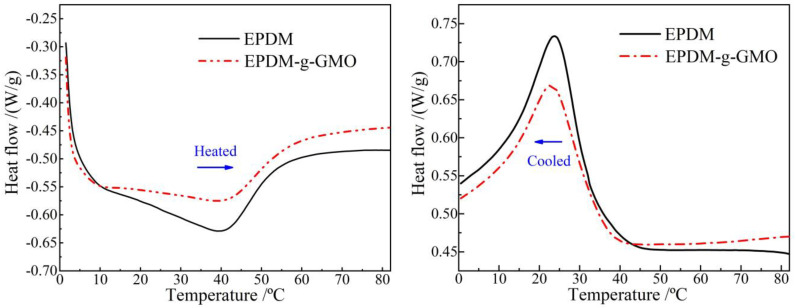
DSC temperature spectra of heat flow in heating (**left panel**) and cooling (**right panel**) processes for the crosslinked EPDM and EPDM-g-GMO materials.

Glassy–elastomeric phase transitions of EPDM and EPDM-g-GMO are represented by endothermic and exothermic heat-flows of DSC temperature spectra in inverse heated and cooled processes as shown in Figure 4 and Table 3. The grafted GMO on EPDM molecular-chains results in a slight increase/decrease in heat-flow peak temperature for phase transition from glassy/elastomeric to elastomeric/glassy states and causes a considerable reduction in phase transition enthalpy, as indicated by integral areas of heat-flow peaks.

The polar-groups on the grafted GMO molecules provide dipoles into EPDM, which increases the dipole moment interaction between EPDM molecular-chains and, thus, enhances the restriction on thermal motions of molecular-chains. Therefore, the grafted GMO accounts for restraining phase transition process in EPDM material, as manifested by the increased difference between two phase transition temperatures in heating and cooling processes and by the substantial abatement of phase transition enthalpy (heat-flow peak magnitude).

**Figure 5:**
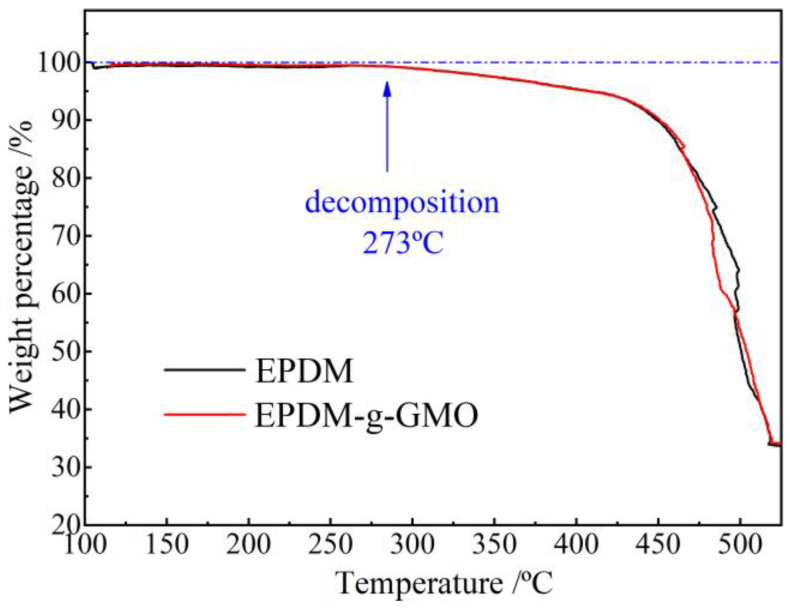
TGA profiles of weight loss versus temperature for the crosslinked EPDM and EPDM-g-GMO materials.

As shown by TGA profiles of EPDM and EPDM-g-GMO in Figure 5, it is evident that EPDM and EPDM-g-GMO almost represent an identical mass loss process with a decomposition temperature of 273 °C, indicating the added GMO component cannot be evaporated out of EPDM in the heating process until EPDM decomposition occurs. It is hereby confirmed by TGA with infrared spectroscopy that the successful graft of GMO molecules onto EPDM molecular-chains is realized by crosslinking process.

### 3.3. Electric Conduction Characteristics

**Figure 6:**
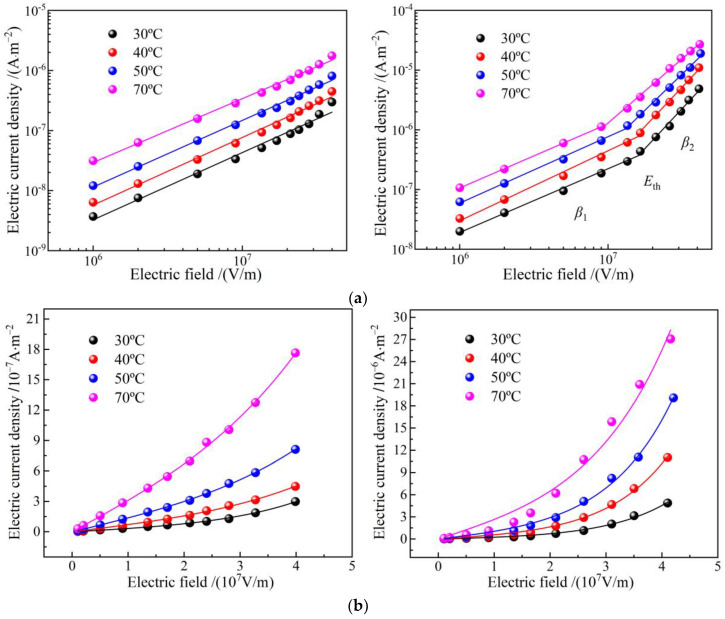
Electric conduction *J-E* characteristics of EPDM (**left panels**) and EPDM-g-GMO (**right panels**) at diverse temperatures from 30 to 70 °C in (**a**) double logarithm coordinates curves for stepwise linear fitting and (**b**) double linear coordinates for hopping conductance fitting.

Characteristic profiles of electric current density versus electric field density (*J*-*E* curves) in double-logarithmic coordinates represent a two-stage linearity, as shown in Figure 6a. The linearly fitted slopes *β*_1_ and *β*_2_ are called linear and nonlinear coefficients, respectively, and the position of crossing point by the two linearly fitted lines is called critical electric field *E*_th_, which arises merely in EPDM-g-GMO rather than EPDM benchmark, as shown by the fitted values in Table 4. As the electric field is raised, the electric conductance transits from Schottky injected conduction (*β*_1_ ≈ 1) to space charge limited conduction (*β*_2_ > 2) in EPDM-g-GMO, which is identified just by *E*_th_ and indicates the trapping–detrapping behavior in charge carrier transport due to GMO graft modification.

Electric current density of EPDM-g-GMO is evidently higher than that of EPDM. Meanwhile, critical electric field and nonlinear coefficient of EPDM-g-GMO are notably reduced as temperature is elevated, which is a manifestation of charge hopping transport (percolation electric conductance) of a thermal excitation process under high electric fields. According to percolation conductance model, the *J-E* curves in linear coordinates, as shown in Figure 6b, are fitted by the following equation [19]:

(1) $$J_{n}=2nd\upsilon e\exp(-\frac{\chi }{k_{b}T})\sin h(\frac{elE}{2k_{b}T})$$

where *n* denotes carrier concentration, *χ* symbolizes activation energy, *l* represents hopping distance of charge carrier, *T* is thermodynamic temperature. The fitting curves are consistent with the tested points of the electric current density for EPDM-g-GMO. As shown by the fitted results in Table 5, the hopping distance decreases with the increase of temperature, implying that the charge detrapping by thermal excitation is exacerbated to expedite hopping transport for both EPDM and EPDM-g-GMO, even mitigating the conductivity nonlinearity of EPDM-g-GMO. Since trapping and detrapping processes are concomitant in a dynamical equilibrium [20], the critical electric field giving rise to a nonlinear field dependence of electric conduction is also an indication of percolation threshold, which is relying on thermal excitation temperature of the trapped charges.

### 3.4. Charge Trap Characteristics

**Figure 7:**
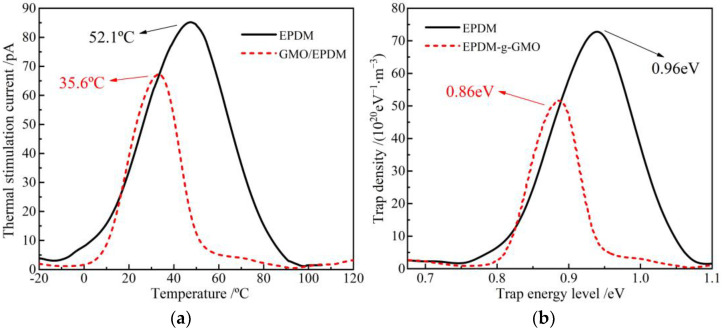
(**a**) TSC temperature spectra and (**b**) trap characteristics of EPDM and EPDM-g-GMO.

TSC temperature spectra and the derived trap energy level distributions of EPDM and EPDM-g-GMO are shown in Figure 7. GMO graft causes charge release peak to shift towards a lower temperature, as indicated by the magnitude-reduced TSC peak of EPDM-g-GMO at the lower temperature of 35.6 °C, compared with EPDM, indicating that charge traps introduced by grafting GMO are shallower in energy level and smaller in density than intrinsic charge traps of EPDM. EPDM-g-GMO represents a shallower trap density peak at 0.86 eV than 0.96 eV of EPDM, indicating a shorter capturing time and a lower scattering probability of charge traps with charge carriers, resulting in a higher charge carrier mobility. Chemical graft of GMO increases electric conductivity of EPDM, which confirms the previous recognition that the amount of charge traps is inversely proportional to electric conductivity in polymer insulation materials [20].

**Figure 8:**
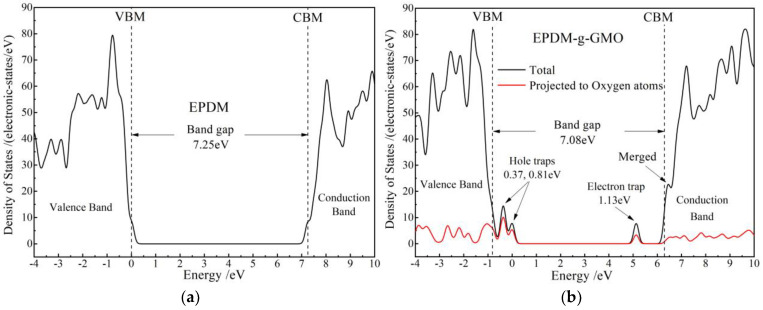
Densities of electronic states of (**a**) EPDM and (**b**) EPDM-g-GMO polymeric molecules.

Energetic distribution density of electronic-states is calculated with first-principles method for EPDM and EPDM-g-GMO to study the effect of graft-introduced electronic bound states on charge traps of EPDM, as shown in Figure 8. In reference to the band-gap of EPDM approaching 7.25 eV, the grafted GMO introduces the electronic bound state (majorly derived from the oxygen of carbonyl) with an almost identical energy level to conduction band minimum (CBM) of EPDM, which becomes the higher density CBM of EPDM-g-GMO and results in a lower band-gap of 7.08 eV than that of EPDM, implying a higher electron mobility at CBM in consistence with experimental results of electric conductance. Moreover, the grafted GMO introduces two shallow hole traps in 0.37 eV and 0.81 eV depths and one deep electron trap of 1.13 eV, majorly deriving from carbonyl oxygen of GMO, which can inhibit space charge accumulation and will mitigate insulation degradation caused by electric conductivity increasing.

### 3.5. Dielectric Breakdown Strength

**Figure 9:**
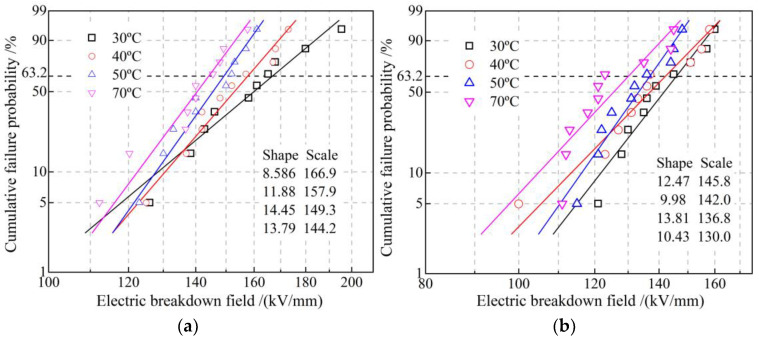
DC electric-breakdown fields fitted in Weibull statistics of (**a**) EPDM and **(b**) EPDM-g-GMO at diverse temperatures from 30 to 70 °C.

Dielectric breakdown strength can be directly evaluated by Weibull distribution scale parameter (characteristic breakdown field with a failure probability of 63.2%), while shape parameter characterizes the stability of dielectric breakdown resistance, as shown in Figure 9. Compared with EPDM, the characteristic breakdown field of EPDM-g-GMO decreases by 12.5% and 13.75 at 30 °C and 70 °C, respectively. According to the first-principles electronic properties of energy-gap *E*_g_, the ionization potential (IP) and electron affinity (EA) for EPDM polymer and GMO molecule as listed in Table 6, the narrower *E*_g_ and higher IP of GMO molecule than that of EPDM polymer favor electron impact ionization (partial discharge) around the grafted GMO, the thermal effect of which will break C-H bonds in EPDM molecular-chains to form carbonized conductive channels accounting for insulation failures. However, the uniformly distributed shallow charge traps introduced by the grafted GMO can inhibit charge injections to mitigate the dielectric breakdown strength reductions also caused by percolation conductance and impact ionization of GMO. Therefore, dielectric breakdown strength of EPDM-g-GMO persists on a sufficiently qualified level for the reinforce insulation in cable accessories.

### 3.6. Electric Field in Cable Terminal

**Figure 10:**
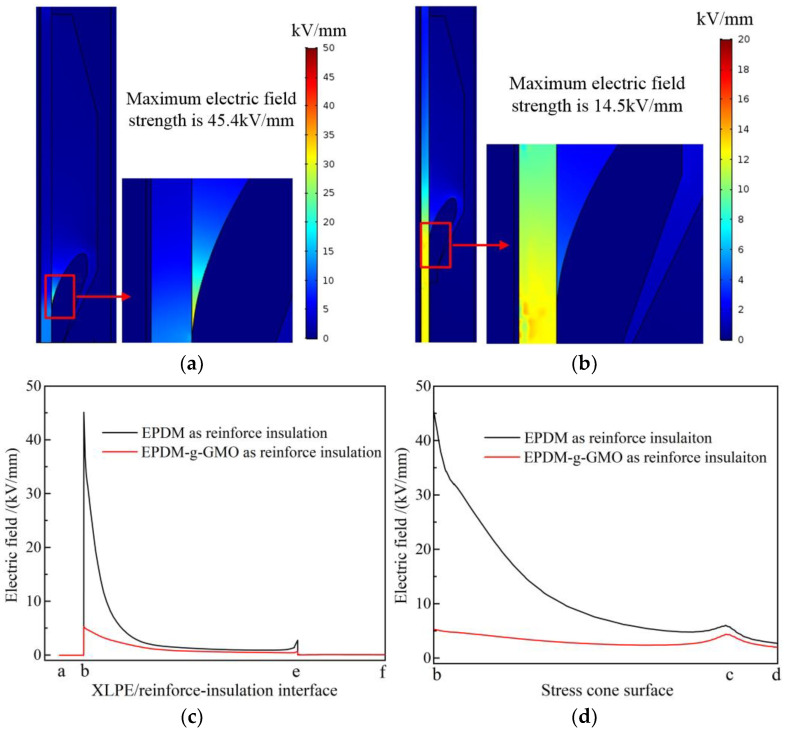
DC steady-state electric fields in cable terminals with (**a**) EPDM and (**b**) EPDM-g-GMO as reinforce insulation, and the tangential electric field varying along (**c**) XLPE/reinforced-insulation interface and (**d**) stress cone surface.

Electric fields of cable terminals individually with EPDM and EPDM-g-GMO as reinforce insulation, being specified by the experimentally fitted electric conductivity versus electric field and temperature, are simulated with finite-element numerical solving method, as shown in Figure 10a,b. In EPDM cable terminal, the maximum electric field approaches 45.4 kV/mm residing at stress cone root. In contrast, the remarkably lower maximum electric field of 14.5 kV/mm in EPDM-g-GMO cable terminal resides inside XLPE main insulation. Further considering the dominant tangential component of electric field along XLPE/reinforced-insulation interface and stress cone surface, as shown in Figure 10c,d, the electric field strength at stress cone root of EPDM-g-GMO cable terminal approaches 5 kV/mm, which is remarkably lower by almost an order of magnitude than that of EPDM cable terminal.

**Figure 11:**
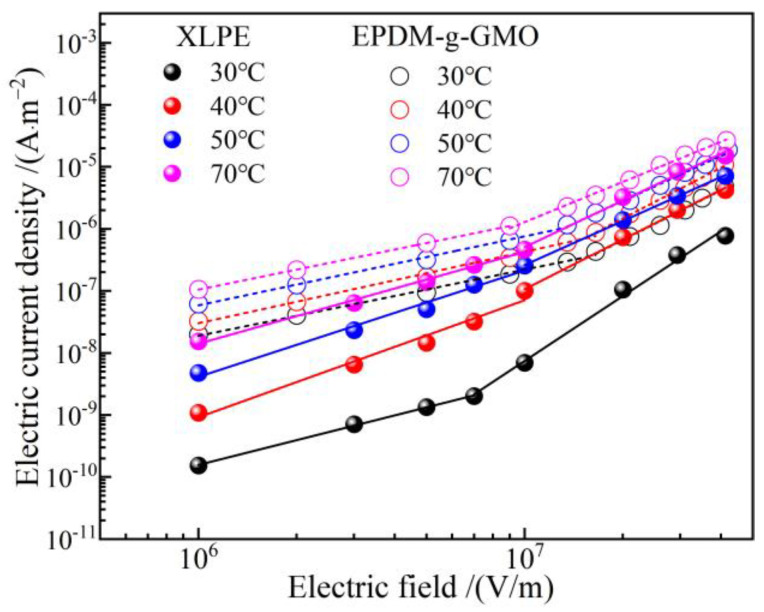
Electric conduction *J-E* characteristics of XLPE compared with EPDM-g-GMO.

Under electrostatic field, the dielectric permittivity of individual cable terminal components made from dielectric materials determines background strengths of internal electric fields, as visually described by electric capacitance in series. More importantly, according to DC electric field distribution principle as recognized classically by electric resistances in series, the intensities of electric fields distributed in every dielectric components of a cable terminal are inversely proportional to the electric conductivity at each point, which is dependent on the thermal field (temperature distribution) derived from material thermal properties in the cable terminal. As shown in Figure 11, the electric conductivity of EPDM-g-GMO is significantly higher than that of XLPE under every DC electric field at various temperatures for cable terminal operations, which cannot be achieved by pure EPDM material. Therefore, by using EPDM-g-GMO as reinforce insulation of a cable terminal, the maximum electric field will arise in XLPE main insulation layer, which is preferred rather than in the reinforce insulation layer of using pure EPDM material. By using EPDM-g-GMO as reinforce insulation, the electrical matching between reinforce insulation and XLPE main insulation can be significantly improved to restrict the maximum electric field into main insulation and effectively homogenize electric field in cable terminals.

## 4. Conclusions

Chemical modification of grafting GMO molecules on EPDM material is realized to improve electric conductance and achieve conductivity nonlinearity, which is suggested to be exploited for fulfilling the highly matching electrical performances between reinforce insulation and main insulation in cable terminals, meanwhile, maintaining a sufficient dielectric breakdown strength for reinforce insulation. First-principles calculations and thermal stimulation current analyses are combined to reveal the charge traps introduced into EPDM by GMO graft, which accounts for the expected modifications in electric conductance and dielectric breakdown. According to the tested results of electric conduction, the GMO-grafted EPDM (EPDM-g-GMO) is specified as reinforce insulation to model cable terminal and calculate electric field with finite-element numerical method, which is performed to demonstrate the effectiveness of GMO-graft modification on EPDM material for homogenizing electric fields in cable accessories. The considerable amount of shallow charge traps introduced by GMO-graft into EPDM are an offset of promoting carrier transports and alleviating the inevitable reduction in dielectric breakdown strength caused by the electric conductivity improvement. The smaller energy-gap and higher ionization potential of GMO molecule than that of EPDM polymer favors electron impact ionization, leading to the exacerbation in dielectric breakdown, while shallow charge traps introduced by GMO graft can inhibit charge injections from electrodes to make EPDM-g-GMO persisting a qualified dielectric breakdown strength for reinforce insulation. When EPDM-g-GMO material is used as reinforce insulation in a cable terminal, the maximum electric field always resides in main insulation, and the maximum electric field in reinforce insulation is much lower than the electric breakdown field of EPDM-g-GMO material.

## Figures and Tables

**Table 1:** Dimensions of individual components in cable terminal.

Components	Dimension/mm
Core radius	19
XLPE thickness	16
Reinforced insulation thickness	64
Shield layer thickness	1
Axial length of stress cone	160

**Table 2:** Material properties specified in electric-thermal coupling simulations.

Materials	Density /(g·cm^−3^)	Relative Permittivity	Thermal Conductivity Coefficient /(W·m^−^^1^·K^−^^1^)	Heat Capacity /(J·kg^−1^·K^−1^)
XLPE	0.91	2.27	1640	0.285
Inner Shield	0.95	100.00	2500	0.510

**Table 3:** Thermal parameters of glassy–elastomeric phase transitions from DSC spectra.

Material	Heat-Flow Peak Temperature /°C	
	Heated	Cooling
EPDM	39.36	23.73
EPDM-g-GMO	39.51	22.73

**Table 4:** Electric conduction characteristic parameters of EPDM-g-GMO at various temperatures.

Temperature /°C	*E*_th_ /(MV/mm)	*β*_1_	*β*_2_
30	17.5	1.01	2.66
40	16.5	1.11	2.62
50	13.8	1.12	2.41
70	9.5	1.05	2.15

**Table 5:** Hopping distance of percolation conductance in EPDM and EPDM-g-GMO.

Temperature /°C	Hopping Distance /nm	
	EPDM	EPDM-g-GMO
30	3.34	4.82
40	2.36	4.73
50	2.27	4.67
70	2.23	3.83

**Table 6:** Electronic properties of EPDM polymer and GMO molecule.

Molecule	*E*_g_ /eV	IP /eV	EA /eV
EPDM	7.25	7.05	−1.45
GMO	5.47	8.03	−0.98

## Data Availability

Theoretical and experimental results are available from the corresponding author.
